# Long-term effects of complications and vascular comorbidity in idiopathic normal pressure hydrocephalus: a quality registry study

**DOI:** 10.1007/s00415-017-8680-z

**Published:** 2017-11-29

**Authors:** Kerstin Andrén, Carsten Wikkelsö, Nina Sundström, Simon Agerskov, Hanna Israelsson, Katarina Laurell, Per Hellström, Mats Tullberg

**Affiliations:** 1 Hydrocephalus Research Unit, Department of Clinical Neuroscience, Institute of Neuroscience and Physiology, The Sahlgrenska Academy, University of Gothenburg, Blå Stråket 7, 413 45 Gothenburg, Sweden; 2 Department of Radiation Sciences, Biomedical Engineering, R&D, Umeå University, 901 85 Umeå, Sweden; 3 Department of Pharmacology and Clinical Neuroscience, Umeå University, 901 87 Umeå, Sweden

**Keywords:** Hydrocephalus, Normal pressure, Dementia, Gait, Complications, Concomitant disease, Risk factors, Registries

## Abstract

**Background:**

There is little knowledge about the factors influencing the long-term outcome after surgery for idiopathic normal pressure hydrocephalus (iNPH).

**Objective:**

To evaluate the effects of reoperation due to complications and of vascular comorbidity (hypertension, diabetes, stroke and heart disease) on the outcome in iNPH patients, 2–6 years after shunt surgery.

**Methods:**

We included 979 patients from the Swedish Hydrocephalus Quality Registry (SHQR), operated on for iNPH during 2004–2011. The patients were followed yearly by mailed questionnaires, including a self-assessed modified Rankin Scale (smRS) and a subjective comparison between their present and their preoperative health condition. The replies were grouped according to the length of follow-up after surgery. Data on clinical evaluations, vascular comorbidity, and reoperations were extracted from the SHQR.

**Results:**

On the smRS, 40% (38–41) of the patients were improved 2–6 years after surgery and around 60% reported their general health condition to be better than preoperatively. Reoperation did not influence the outcome after 2–6 years. The presence of vascular comorbidity had no negative impact on the outcome after 2–6 years, assessed as improvement on the smRS or subjective improvement of the health condition, except after 6 years when patients with hypertension and a history of stroke showed a less favorable development on the smRS.

**Conclusion:**

This registry-based study shows no negative impact of complications and only minor effects of vascular comorbidity on the long-term outcome in iNPH.

## Introduction

Idiopathic normal pressure hydrocephalus (iNPH) is a treatable disorder, presenting with gait and balance difficulties, cognitive impairment and urinary incontinence, and has a prevalence of 1.4–2% in the elderly [1, 2]. INPH is treated by insertion of a CSF-diverting shunt system, which improves more than 80% of the patients on a short-term basis [3–5].

There are several studies on the long-term outcome after shunt surgery for iNPH patients, showing a favorable outcome after 3–7 years in 26–91% of the patients [6–20]. On the basis of five of these studies, published in 2006–2010, a systematic review by Toma et al. concluded that 73% of the patients still benefit from shunt surgery after 3 years or more [3].

The same review found a revision rate of 13%, subdural hematomas in 4.5% and infections in 3.5% of the patients, and a shunt surgery-related mortality rate of 0.2% [3]. Pujari et al. showed that 74% of the patients improved after shunt revision [10], but the long-term effects of shunt revision have not been described.

INPH patients have a heavier burden of hypertension [21–25], diabetes mellitus [22–27], hyperlipidemia [27], obesity [27], ischemic heart disease [22–24] and arteriosclerotic cerebrovascular disease [22] than control groups. Several findings indicate that risk factors for arteriosclerotic cerebrovascular disease are involved in the pathogenesis of iNPH [21, 28], and it has recently been suggested that iNPH may be a subtype of vascular dementia [27].

Earlier studies show that a good shunt response is also seen in iNPH patients with extensive presumed ischemic white matter lesions [16, 29], that radiological signs of cerebrovascular disease cannot predict the outcome in iNPH patients [30], and that the magnitude of improvement in patients with and without vascular comorbidities is the same [4]. However, other studies report contrasting findings, showing less improvement in patients with signs of ischemic cerebrovascular disease [31, 32]. There is little information regarding the effects of vascular risk factors and comorbidities on the long-term outcome in iNPH patients [16, 33].

The Swedish Hydrocephalus Quality Registry (SHQR) is the largest national registry for adult hydrocephalus patients including prospectively collected clinical data. A recent report, based on SHQR data, described the incidence of hydrocephalus surgery in Sweden during 2004–2011 and the 3-month outcome for iNPH patients [34], but no long-term evaluations have so far been reported.

The aim of this study was to describe the long-term outcome of iNPH patients included in the SHQR, the incidence and influence of reoperation due to complications, and the influence of vascular risk factors and vascular comorbidity on outcome.

## Methods

The 979 patients diagnosed and treated for iNPH during 2004–2011 and registered in the SHQR before September 2014 were included. The patients were registered at five of the six neurosurgical centers in Sweden: the University Hospitals in Umeå (*n* = 119), Uppsala (*n* = 313), Linköping (*n* = 164), Gothenburg (*n* = 148), and Lund (*n* = 235). During this period, the sixth neurosurgical center in Sweden did not report to the SHQR.

The treatment was shunt surgery in 974 patients (ventriculo-peritoneal: 953, ventriculo-atrial: 6, not specified: 15 patients), and 5 patients were operated by a third ventriculostomy; 3 of whom were later reoperated with shunt insertion (after 2 weeks, 6 weeks and 8 months, respectively).

The diagnosis of iNPH was made according to each center’s routines, based on the criteria for possible or probable iNPH [35] in the International guidelines.

Information concerning baseline characteristics, including the presence of hypertension, diabetes mellitus, heart disease and history of stroke (Table 1), the date and type of primary surgery and reoperations, preoperative and 3-month postoperative clinical evaluations, along with long-term evaluations from postal follow-ups, was extracted from the SHQR on the 1st of September 2014.

**Table 1:** Baseline characteristics for 979 patients diagnosed and operated on for iNPH during 2004–2011, included in the Swedish Hydrocephalus Quality Registry, SHQR

Demography	iNPH patients, *n* = 979
Age (years), median (IQR)	74 (68–78)
Sex, female, *n* (%)	413 (42)
Preoperative modified Rankin Scale (mRS), median (IQR)	2 (2–3)

Vascular comorbidity	*n* (%)
Hypertension	438 (49)
Diabetes mellitus	189 (21)
History of stroke	119 (14)
Heart disease	231 (26)

Number of vascular comorbidities	*n* (%)
0	401 (41)
1	279 (28)
2	175 (18)
3	65 (6.6)
4	8 (0.8)
Not available	51 (5.2)

*INPH* idiopathic normal pressure hydrocephalus, *IQR* interquartile range, *mRS* modified Rankin Scale

The preoperative and the 3-month postoperative clinical evaluations included the modified Rankin Scale (mRS) [36], with scores of 0–5 [37] (Table 2).

**Table 2:** mRS^37^ and smRS scales, the latter translated from Swedish. The mRS was used in clinical evaluations pre- and 3 months postoperatively. The smRS was used for patient’s self-assessment of their degree of independent daily function or disabilities in the long-term evaluations

	Modified Rankin Scale, mRS	Self-assessed modified Rankin Scale, smRS
		Headline in the questionnaire: “Disability/need for assistance”
0	No symptoms at all	No problems
1	No significant disability despite symptoms: able to carry out all usual duties and activities	Some problems that do not restrict my lifestyle
2	Slight disability: unable to carry out all previous activities but able to look after own affairs without assistance	Minor disability, some restrictions to my lifestyle, no need for assistance
3	Moderate disability: requiring some help, but able to walk without assistance	Some disability, which clearly restricts my lifestyle, need for assistance
4	Moderately severe disability: unable to walk without assistance, and unable to attend to own bodily needs without assistance	Severe disability, dependent but not in constant need of assistance
5	Severe disability: bedridden, incontinent, and requiring constant nursing care and attention	Very severe disability, in need of constant care, day and night

At the 3-month postoperative visit, the physician assessed if the patients’ general health condition was “better”, “unchanged” or “worse” than before surgery. This variable was included in the SHQR protocol during 2008–2010 and ratings were available for 177 patients.

### Long-term outcome

The outcome measures used for the long-term follow-up were a self-assessed mRS (denoted smRS, Table 2), and an enquiry of the present health condition in comparison with before surgery, phrased: “How are you feeling now, compared with your condition before surgery?” with three response alternatives: “better”, “unchanged” or “worse”.

The standardized questionnaires for the long-term follow-up had initially been mailed annually to the patients starting from 2 years after surgery. In 2010, the routine was changed to sending out questionnaires after 2, 5 and 10 years. A cover letter explained the purpose and asked that the questionnaire be completed at home, by the patient, a next-of-kin or caretaker. A reminder was sent to patients who did not reply to the first letter.

Of the 979 patients, 623 (64%) returned 1–6 questionnaires (median 1, IQR 1–2) and the time interval from surgery to returned questionnaires ranged from 1.6 to 10.5 years (median 3, IQR 2.3–4.5). The questionnaire replies were sorted into groups, as shown in Table 3. All responses from the patients are included in the respective groups, except for two patients who replied twice during the same year; only one of these replies from each patient was included.

**Table 3:** Replies to questionnaire on long-term evaluation of 979 iNPH patients operated on in Sweden in 2004–2011. Available patients at each year after surgery were defined as living patients with follow-up within each time range

Group	Time interval (years)	Returned questionnaires (*n*)	Proportion of available patients (%)
Two years	1.6–2.49	297	33
Three years	2.5–3.49	247	29
Four years	3.5–4.49	150	23
Five years	4.5–5.49	144	32
Six years	5.5–6.49	56	17
Seven years	6.5–7.49	18	8
Eight years	7.5–8.49	2	1
Nine years	8.5–9.49	4	4
Ten years	9.5–10.5	5	13
Total		923	
Ever replying patients		*n* = 623	Proportion 64%

Only follow-up at 2, 3, 4, 5 and 6 years were presented and analyzed, due to the scarcity of responses after 7–10 years (Table 3).

Three hundred and fifty-six patients were lost to long-term follow-up. Sixty-seven of these were deceased at the time of the 2-year letter. Thirty-one patients declined participation.

### Complications and reoperations

The occurrence of complications and reoperations was continuously registered in the SHQR, thus available up to 10 years after the initial surgery. The influence of reoperation on the outcome was studied up to 6 years after surgery.

Only complications leading to reoperation were counted and grouped into (1) mechanical: most commonly, shunt obstruction or displacement; (2) infections: intraabdominal, skin or CNS infections; (3) subdural hematomas (evacuation of hematoma and/or ligation of shunt), and (4) other or not specified. All reoperations were registered separately even if they referred to the same complication; for instance, management of an intraabdominal infection, first by externalization of the distal catheter and later by removal of the whole shunt, would count as two operations. However, shunt insertions performed after ventriculostomy, reinsertions after earlier shunt removals, and reopening of temporarily ligated shunts were not considered related to new complications and, therefore, not registered in this study.

All patients received written information about inclusion in the register, and that they could withdraw from participation at any time.

The study was approved by the Regional Ethical Review Board in Gothenburg (Reg. no. 492-14, with addition T006-15).

### Statistics

Only non-parametric tests were used. Proportions were compared using Fisher’s exact test. For comparisons between groups, the Mann–Whitney *U* test was used, and for within-group comparisons, the Wilcoxon signed rank test was used. To analyze the influence of the vascular comorbidities on long-term outcome, odds ratios with a 95% confidence interval were calculated by logistic regression analysis adjusted for age and sex. Correlations were tested by the Spearman rank correlation. All significance tests were two tailed and the statistical significance was set at the 0.05 level. All analyses were performed with SPSS 24.0 (IBM, Armonk, New York, USA).

## Results

Three months after surgery, 39% of the patients had improved ≥ 1 step on the mRS. Two, 3, 4, 5 and 6 years after surgery, 41, 41, 38, 40 and 40%, respectively, were improved ≥ 1 step on the smRS, compared with the baseline mRS.

**Fig. 1:**
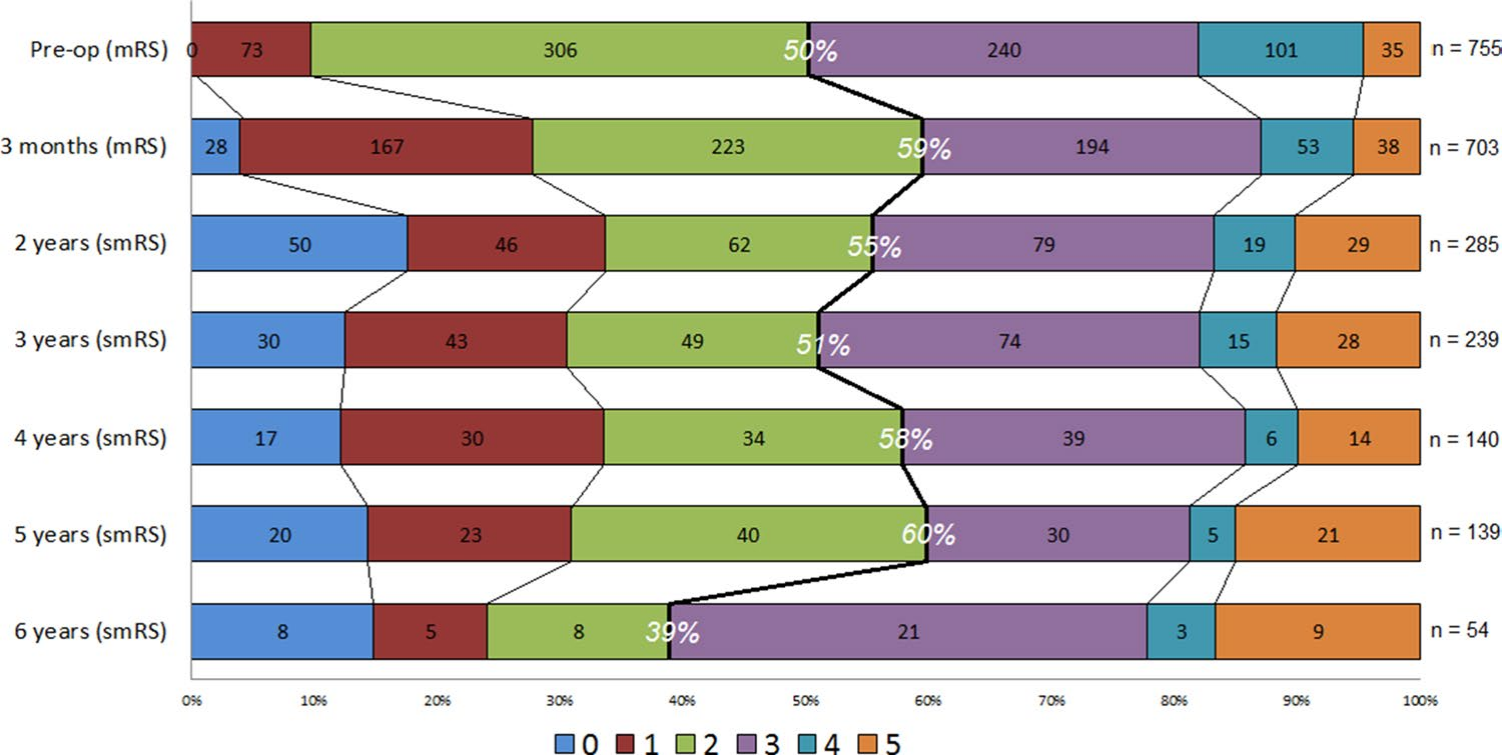
Shift analysis of modified Rankin Scale (mRS) scores (0–5) in 979 iNPH patients: at baseline and 3 months after surgery, and self-reported mRS (smRS) at 2–6 years after surgery. Black numbers within the bars represent the number of patients obtaining each score on the mRS and smRS. White numbers show the proportion of patients with scores between 0 and 2, i.e., able to live a life independent of help from others

The patients were improved on the mRS at the 3-month evaluation (median 2, IQR 2–3 preoperatively vs. median 2, IQR 1–3 postoperatively, *p* < .001), and remained improved on the smRS after 2 years (median 2, IQR 1–3, *p* = .001). The smRS at 3, 4, 5 and 6 years were unchanged compared with the mRS preoperatively (median 2–3, *p* = .22–.86) (Fig. 1).

**Fig. 2:**
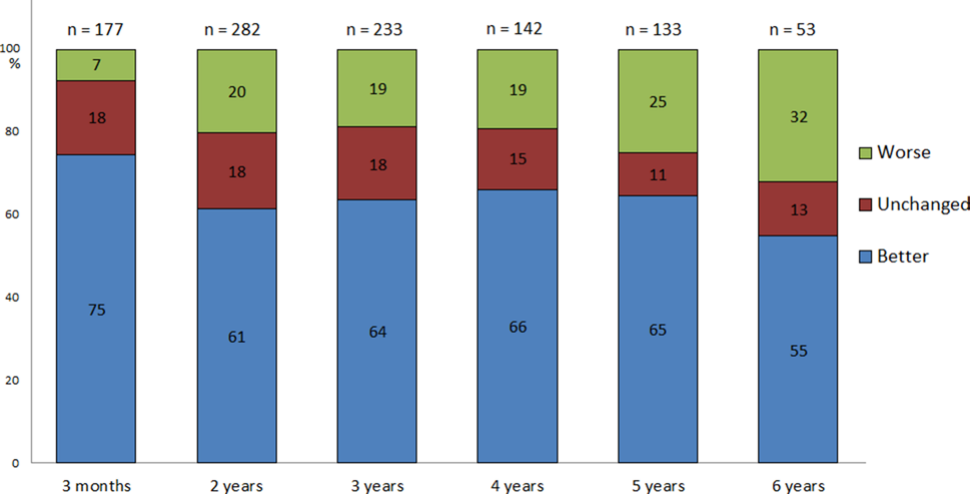
Postoperative health condition in 979 iNPH patients. Percentage of iNPH patients reporting better, unchanged or worse health condition, of whom the following question was asked: “How are you feeling now, compared with your condition before surgery?” after 2–6 years. The 3-month evaluation was carried out in the clinical follow-up setting; results at the 2- to 6-year evaluation come from follow-up questionnaires. The number of available answers at each time point is indicated above the bars

Around 60% of the patients described their health condition as improved after 2–6 years (Fig. 2).

### Reoperations

One or more complications were found in 26% of the patients (257 patients), leading to a total of 410 reoperations. Of these, 58% (239 reoperations) took place during the first year after the primary surgery. Of the 257 patients, 61% (157 patients) had only one reoperation, 26% (66) had two and 13% (34) had three or more reoperations (median 1, range 1–5).

**Fig. 3:**
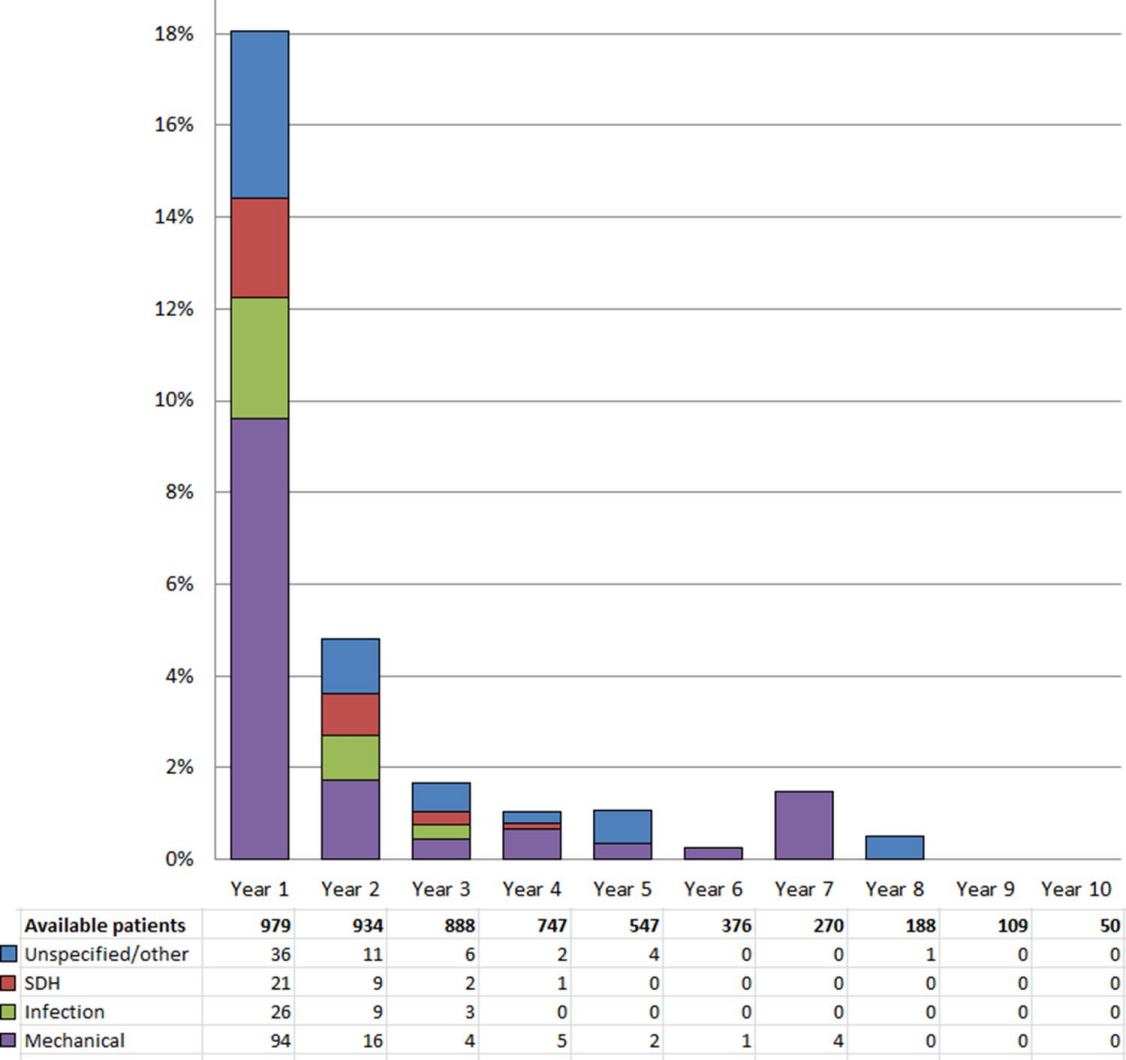
Complications causing first-time reoperations in 979 iNPH patients, visualized year by year after primary surgery. For each year, the percentage of patients who needed reoperation because of complications is represented in the bar chart, in four groups based on the type of complication. The corresponding numbers of patients and the total number of available patients for each year are shown in the table

In total, 14% (141) of the 979 patients had reoperations due to mechanical complications, 6.4% (63) due to infections, 3.7% (36) due to subdural hematomas and 9.1% (89) due to other or not specified causes. These groups are overlapping, as one patient may have had more than one type of complication at different time points. The patients’ first complications leading to reoperation are shown in Fig. 3.

There were no differences in long-term outcome for patients who had or had not been subjected to reoperation (Table 4).

**Table 4:** Outcome 2–6 years after primary surgery in iNPH patients who underwent reoperation, compared with patients in whom reoperation was not performed. The outcome measures are self-reported mRS (smRS) and “Improved health condition”, signifying the proportions of patients replying “better” to the following question: “How are you feeling now, compared with your condition before surgery?”

	Reoperated patients, *n* (% of available questionnaire responses at specified year)	smRS, median (IQR)		*p*	Improved health condition, *n* (%)		*p*
		Re-op.	Not re-op.		Re-op.	Not re-op.	
2 years	53 (18)	2 (1–3)	2 (1–3)	.61	26 (53.1)	147 (63.1)	.20
3 years	55 (22)	3 (1–3)	2 (2–3)	.15	29 (55.8)	119 (65.7)	.20
4 years	39 (26)	2 (1–3)	2 (2–3)	.20	21 (56.8)	73 (69.5)	.16
5 years	42 (29)	2 (2–3)	2 (1–3)	.21	18 (52.9)	68 (68.7)	.15
6 years	12 (22)	3 (1–5)	3 (2–3)	.78	7 (53.8)	22 (55.0)	1.0

*Re-op.* reoperated

The mortality rate related to shunt surgery, defined as death of any cause within 1 month of the surgical procedure, was 0.5% (five patients).

### Vascular comorbidities

Preoperatively, patients with diabetes, stroke or heart disease had higher mRS scores than patients without these factors. Three months postoperatively, patients with stroke or heart disease were still significantly worse (higher mRS), while patients with diabetes were not. Postoperative improvement of the health condition according to physicians’ ratings did not differ between those with or without any of the four comorbidities (Table 5).

**Table 5:** mRS scores before and 3 months after surgery, along with physicians’ rating of postoperative relative to preoperative health condition in iNPH patients with comorbidities, compared to those without

	*n* (% within available scorings at specified time point)	mRS, median (IQR)		*p*	Improved health condition, *n* (%)		*p*
		With	Without		With	Without	
Preoperative							
Hypertension	350 (49.7)	3 (2–3)	2 (2–3)	.75	–	–	–
Diabetes	147 (21)	3 (2–3)	2 (2–3)	.019	–	–	–
Stroke	93 (13.3)	3 (2–4)	2 (2–3)	< .001	–	–	–
Heart disease	171 (24.2)	3 (2–3)	2 (2–3)	.002	–	–	–
3 months							
Hypertension	331 (51.2)	2 (1–3)	2 (1–3)	.36	66 (70)	62 (80)	.16
Diabetes	141 (21.8)	2 (2–3)	2 (1–3)	.082	28 (68)	101 (76)	.41
Stroke	91 (14.2)	3 (2–3)	2 (1–3)	< .001	17 (77)	111 (74)	.80
Heart disease	161 (24.8)	3 (2–3)	2 (1–3)	.013	25 (70)	103 (75)	.52

*mRS* modified Rankin Scale

With regard to the actual smRS ratings, there were no differences between patients with vascular comorbidity and those without, except for patients with a history of stroke in the 3-year group, who had a higher degree of disability (median smRS 3 vs 2, *p* = .024), i.e., similar to the differences found before surgery and at the 3-month evaluation.

Looking instead at the differences between the smRS at the different time points and at baseline, the magnitude of improvement did not differ between patients with and without vascular comorbidity, except for the follow-up after 6 years, where patients with hypertension and patients with a history of stroke both had a less favorable development than those without comorbidity (*p* = .045 for both comparisons).

Regarding the likelihood of patients describing their health condition as “better” as opposed to “unchanged” or “worse”, there were no differences between those with any of the comorbidities and those without.

There were no significant correlations between the number of comorbidities (0–4) and differences between the mRS score at baseline and subsequent smRS scores 2–6 years after surgery, nor with the evaluation of the health condition (“better”, “unchanged” or “worse”) at the same time points (correlation coefficients − .122 to .249).

## Discussion

This study shows lasting improvement on a self-assessed modified Rankin Scale in around 40% of shunted iNPH patients, and lasting subjective improvement in about 60% at follow-up after 2–6 years. The reoperation rate within 10 years was 26%, of which 58% were performed within the first year of surgery. Reoperation did not impede improvement up to 6 years after primary surgery.

Diabetes, heart disease, and a history of stroke were each associated with higher mRS scores at baseline, and the latter also after 3 years. However, neither of these three, nor hypertension, had a negative influence on the degree of postoperative improvement until more than 5 years after surgery.

### Complications

The reoperation rate of 26% for any cause in our data was rather high. Fourteen percent of the patients had revisions due to mechanical problems. In the systematic review by Toma et al., the revision rate, constituting the need for revision due to shunt failure or “mechanical causes”, was 13% in 30 articles published after 2006 [3]. One explanation for a higher reoperation rate in the current material is the longer follow-up period. Most studies have follow-up periods of up to 1 year, while 42% of the revisions in the present study were performed later. Another explanation could be that patients included in other studies aiming to investigate, e.g., specified measurements may have been carefully selected, whereas the present study includes all operated iNPH patients from the participating centers.

SDH was reported in 4.5% of the patients in the review [3], which is in the same range as in our study (3.7%). However, our figure shows only those requiring surgical treatment, and the total number of SDH is probably higher. The infection rate of 6.4% is higher than in many other studies (average 3%, but ranging from 0 to 10%) [3]. Similarly, the operation-related mortality of 0.5% is somewhat higher than in contemporary studies. This was calculated in the cited review to be 0.2%, for the years 2006–2010, with a range between 0 and 0.8%, for the 13 studies reporting numbers on shunt surgery-related mortality [3]. Again, the unselected patient material in the SHQR compared with the less extensive single-center studies with stricter inclusion criteria reviewed by Toma et al. [3] may have influenced the results.

One of the important findings in this study is that the complications leading to reoperation had no negative effects on the long-term outcome. This knowledge is essential for clinicians, well aware of the risk of problems with shunt surgery, when advising patients. Of course, complications cause a temporary decline in physical and mental functions but it appears that identification and treatment prevent major decline.

### Comorbidities

There is increasing evidence that risk factors for cerebrovascular disease contribute to the pathophysiology of iNPH, and a recently published study calculated that multiple vascular risk factors could explain 24% of iNPH cases (population attributable risk, PAR, 24%) [27]. Our study could not show that the specific vascular risk factors of hypertension or diabetes hampered the effects of shunting, either in the short- or long-term perspective, even if patients with diabetes performed worse in the baseline evaluation. This is in line with earlier studies concerning the influence of vascular risk factors on short-term outcome [4, 16, 31], while their influence on the long-term outcome in iNPH patients has not previously been described.

Macro- and microvascular changes, where both hypertension and diabetes are thought to play a role, with stiffening of the arterial walls and development of endothelial dysfunction in small vessels, causing increased permeability [38], could hypothetically contribute also to the disturbed hydrodynamics in iNPH, with increased CSF pulsations [39] and diminished perfusion in small vessels in the periventricular region [40, 41]. Shunting immediately changes the absorption mechanism and thereby “eliminates” both the vascular and hydrocephalic component of the disturbed CSF dynamics, as well as improves periventricular perfusion [42]. As the effect of the shunt is permanent it may even compensate for the deterioration known to be associated with hypertension and diabetes and thereby explain the lack of negative long-term effects.

Earlier studies showed a less favorable outcome for patients with established cerebrovascular disease (CVD), defined as a history of stroke, infarctions on radiological imaging, or moderate to severe white matter lesions on a CT scan [16, 31]. Boon et al. found 52% of the patients with CVD to be improved, as opposed to 79% of those without [31], and Spagnoli et al. showed similar results in the long-term perspective (mean 52 months), with 49 vs. 79% improved patients [16]. Our study included only a history of stroke, but the negative impact of CVD, in this narrower sense, regarding the proportion of improved patients in the short or long term could not be confirmed. Patients with a history of stroke performed worse with regard to the smRS score only after 3 years, but the proportion of patients still reporting “better” was not affected at any time point.

The main limitation to this study is the low letter response rates, ranging from 17% of available patients in the 6-year group to 33% in the 2-year group (Table 3). During some periods, letters were not sent from all centers as intended, which explains the lack of data in long-term follow-ups for 258 patients (26%), and why not all patients were followed up at each pre-specified time point.

However, the incomplete mailings were not influenced by patient characteristics. When comparing patients in each of the long-term follow-up groups to those without replies, there were no significant differences regarding the mRS at baseline or age at surgery, and the frequencies of the four reported comorbidities were equal. Furthermore, the prevalence of reoperation before follow-up in the 3-, 4-, 5- and 6-year groups was similar in repliers and non-repliers. Only in the 2-year group, which was closest in time relative to the majority of the reoperations, repliers had undergone fewer reoperations than non-repliers: 18% compared to 27% (*p* = .004). Altogether, this should indicate that there are no systematic errors in the distribution of follow-up letters, arguing for the representativity and validity of the samples.

The benefits of mailed follow-up questionnaires are that they allowed for follow-up of a large number of patients, and that the responses in the questionnaires are in no way influenced by an examiner.

Another limitation is the use of subjective outcome measures. The smRS and the patients’ comparisons between their present and their preoperative health condition as measures of outcome are evaluations whose correspondence to objective measures has not been studied. Still, we consider these measures valuable, representing outcome as experienced by the affected patient.

The major strength of the study is that it is a quality registry-based study, which allowed for a very large sample size of 979 patients with data collected prospectively during a long follow-up period of up to 10 years. Patients were diagnosed in five different centers, covering approximately 80% of the referral areas in Sweden [34], according to standardized and clinically applied routines representing everyday clinical practice. There were no specific exclusion criteria, such as those often applied in other scientific studies, why the study cohort probably reflects the target population of iNPH patients more accurately.

Based on these results, physicians can inform patients about the risk of complications with greater certainty—stating that it is fairly high, but that complications are treatable and that no negative effect on the long-term outcome has been shown. These findings also give support to decisions regarding patients with hypertension, diabetes, heart disease or stroke, as these extensive data did not show a less favorable outcome for these patients, meaning that they should not be excluded from shunt surgery.

## Conclusion

This registry-based study shows no negative impact of complications and only minor effects of vascular comorbidity on the long-term outcome in iNPH.
